# A new record and a novel morph description of *Boigastoliczkae* (Squamata, Colubridae) from China

**DOI:** 10.3897/BDJ.12.e123669

**Published:** 2024-07-04

**Authors:** Shiyang Weng, Dian-Cheng Yang, Cong Wei, Peng Li, Zhangbo Cui, ZhiHao Jiang, Song Huang

**Affiliations:** 1 Tibet Plateau Institute of Biology, Tibet, China Tibet Plateau Institute of Biology Tibet China; 2 College of Life and Environmental Sciences, Huangshan University, Huangshan, China College of Life and Environmental Sciences, Huangshan University Huangshan China; 3 Huangshan Noah Biodiversity Institute, Huangshan, China Huangshan Noah Biodiversity Institute Huangshan China; 4 Anhui Province Key Laboratory of the Conservation and Exploitation of Biological Resource, College of Life Sciences, Anhui Normal University, Wuhu, China Anhui Province Key Laboratory of the Conservation and Exploitation of Biological Resource, College of Life Sciences, Anhui Normal University Wuhu China; 5 Bureau of Natural Resources of Gyirong County, Tibet, China Bureau of Natural Resources of Gyirong County Tibet China

**Keywords:** Stoliczka´s Asian Cat Snake, Himalaya, distribution, colour and pattern polymorphism

## Abstract

**Background:**

The Asian Cat Snake genus *Boiga* Fitzinger, 1826 includes 37 species, with high species diversity. Five species of *Boiga* have been recorded in China including *B.multomaculata* (Boie, 1827), *B.kraepelini* (Stejneger, 1902), *B.cyanea* (Duméril, Bibron & Duméril, 1854), *B.guangxiensis* (Wen, 1998) and *B.siamensis* (Nutaphand, 1971). Previously, the validity of the species *Boigastoliczkae* (Wall, 1909) was controversial. *B.stoliczkae* was considered in synonymy with *B.ochracea*. Currently, the taxonomy of *B.multomaculata* and *B.ochracea* (Theobald, 1868) was revised so that *B.multomaculata* and *B.ochracea* actually represent a single species and *B.stoliczkae* was recognised as a valid species. *B.stoliczkae* was previously known to be found in the west from central Nepal through Darjeeling, Sikkim and Bhutan to Arunachal Pradesh and Assam in north-eastern India.

**New information:**

One adult female specimen of the Asian Cat Snake was collected from Gyirong County, near the China-Nepal border, Tibet, China during fieldwork on August 2023. We compared morphology and mitochondrial DNA sequence data with all the species of the genus *Boiga*. Both datasets strongly supported referring the Chinese specimens to *B.stoliczkae* (Wall, 1909) due to the 21 mid-dorsal scale rows and the uncorrected *p*-distance (mitochondrial DNA gene cytochrome b) between this specimen and *B.stoliczkae* which is 1.7%. We further described morphological characters of the Chinese specimen in detail and compared these with the specimens that had been previously described. The dorsal ground colour of the Chinese specimen is dark brown, with a black stripe distributed almost evenly across the tail. This is a novel morph of the species *B.stoliczkae*. The newly-collected Chinese specimen expands the distribution of the species on the Himalaya range.

## Introduction

The colubrid snake genus *Boiga* Fitzinger, 1826 includes 37 species, primarily arboreal snake species and these are distributed from the Southwest and Central Asia, South and Southeast Asia, many islands in the western Pacific to northern Australia, with particularly high species diversity in Southeast Asia (Uetz et al. 2024). Colour polymorphism has been previously reported in several colubrid snakes including *Boiga* spp. Characters of colouration and patterning are also utilised in the available dichotomous keys to the genus *Boiga* (Giri et al. 2019, Ganesh et al. 2020). The colour polymorphism has caused great confusion in the taxonomy of the genus *Boiga*.

Previously, the validity of the species *B.stoliczkae* (Wall, 1909) was controversial (Wall 1909, Smith 1944, Kramer 1977). *Wall (1909)* examined 39 specimens from the Darjeeling area of West Bengal, India, which exhibited 21 dorsal scale rows, 218–252 ventrals and 100–119 subcaudals and described those specimens as a new species, named as *Dipsadomorphusstoliczkae*. Smith (1944) synonymised *B.stoliczkae* with *B.ochracea*. Kramer (1977) did not synonymise *stoliczkae* with *ochracea*, but rather recognised it as a subspecies, *B.ochraceastoliczkae*. Currently, the taxonomy of *B.multomaculata* and *B.ochracea* (Theobald, 1868) has been revised, the analyses of molecular genetic data and morphological data providing evidence that the Asian cat snake taxa *B.multomaculata* and *B.ochracea* actually represent a single species and *B.stoliczkae* is now recognised as a valid species and is formally resurrected from synonymy with *B.ochracea* (Köhler et al. 2023). The species *B.stoliczkae* is known to be found in the west from central Nepal through Darjeeling, Sikkim and Bhutan to Arunachal Pradesh and Assam in north-eastern India (Köhler et al. 2023).

Five species of *Boiga* have been recorded in China including *B.multomaculata* (Boie, 1827), *B.kraepelini* (Stejneger, 1902), *B.cyanea* (Duméril, Bibron & Duméril, 1854), *B.guangxiensis* (Wen, 1998) and *B.siamensis* (Nutaphand, 1971) (Zhao 2006, Huang et al. 2021). During a herpetological survey in Gyirong County, near the China-Nepal border, Tibet, China on 31 August 2023, we collected one specimen of *Boiga*. Based on morphometric characters and molecular genetic data, we identified it as *B.stoliczkae*. This finding not only adds to our understanding of the biodiversity in this remote region, but also highlights the importance of continuous monitoring and research in such ecologically sensitive areas. Moreover, the significance of our study lies in the reinforcement of the need for biodiversity surveys, inventories and conservation efforts for herpetofauna in general and, specifically, for this species. Biodiversity surveys play a crucial role in documenting the presence and distribution of species, while inventories provide vital information on population sizes and trends. These efforts are instrumental in identifying conservation priorities and developing effective management strategies to safeguard the ecological integrity of these fragile ecosystems. As our findings demonstrate, even in seemingly remote and unexplored regions, new discoveries can be made that further our understanding of the natural world and the need for its protection.

## Materials and methods

**Sampling.** The specimen was collected in the field using visual surveys. Liver and muscle tissues were extracted and immediately preserved in 95% ethanol. The specimen was fixed in 10% formaldehyde for one day, then transferred to 75% ethanol for permanent preservation and deposited in the Tibet Plateau Institute of Biology (collection number: HSR23050, voucher number TBR2023045). The animal study protocol was approved by the Laboratory Animal Care and Animal Ethics Committee of Tibet Plateau Institute of Biology. Furthermore, with the explicit permission and approval of the Tibet Autonomous Region Forestry and Grassland Administration, all sampling activities were conducted. We not only obtained the necessary permits, but also adhered to strict ethical guidelines, ensuring full compliance with local regulations and minimising any potential impact on the natural habitat.

**Morphological examination.** The number of ventral scales was counted according to Dowling 1951a. Dorsal scale row reduction formulae were based on Dowling 1951b. Morphometrics measurements and other scale counts were carried out following Zhao 2006 and Huang 2021. Body and tail length measurements were taken with a ruler to the nearest 1 mm. All other measurements were made using digital calipers to the nearest 0.1 mm.

Abbreviations for measurements and scale characters are following Darko et al. 2022. The measurements and their abbreviations are as follows: snout-vent length (SVL): measured from the tip of the snout to the anterior edge of the vent; tail length (TAL): measured from the anterior edge of the vent to the tip of the tail; total length (TL) defined as the sum of SVL and TAL; head length (HL): taken from the tip of snout to the posterior margin of mandible; head width (HW): measured from the widest part of the head in dorsal view; eye diameter (ED): taken from the most anterior corner of the eye to the most posterior corner. The scale characters and their abbreviations are: maxillary teeth (MT), supralabials (SL), infralabials (IL), loreals (LOR), preoculars (PRO), postoculars (PO), Chin, infralabials touching the first pair of chin shields (IL-1st Chin), temporals (TEMP), supraoculars (SPO), three dorsal scale row (DSR) counts: 1) counting from one head length behind the head, 2) at mid-body and 3) at one head length before the vent; ventral scales (VS), cloacal plate (CP) and subcaudal (SC). The specimen was compared with other species of the genus *Boiga*, based on descriptions in the available literature (Zhao 2006, Köhler et al. 2023).

**Molecular phylogeny.** Genomic DNA was extracted from liver tissue using a Qiagen DNEasy blood and tissue extraction kit (Qiagen Inc., Valencia, CA). The partial mitochondrial DNA gene encoding cytochrome b (cyt b) was obtained by polymerase chain reaction (PCR) using primer pairs L14910 (5’-GAC CTG TGA TMT GAA AAC CAY CGT TGT-3’) and H16064 (5’-CTT TGG TTT ACA AGA ACA ATG CTT TA-3’) (Burbrink et al. 2000). PCR products were sequenced by Shanghai Map Biotech Co. Ltd. and raw sequences were assembled using seqman in the DNAStar software package (Burland 2000). The newly-generated sequence was deposited in GenBank (Accession number: PP431561).

All sequences were aligned and compared with each other separately on the same gene loci by MEGA X software (Tamura et al. 2021). ModelFinder was used to select the best-fit model using BIC criterion (Kalyaanamoorthy et al. 2017). The best-fit model according to BIC is : GTR +FO +G4m. ML phylogenetic analyses were inferred, based on these alignments by RAxML-NG 1.0.0 with the GTR +FO +G4m model. Each inference was initiated with a random starting tree and a majority rule consensus tree was calculated with 1000 bootstrap replicates.

## Data resources

All the cyt b sequences in this study were retrieved from GenBank and uncorrected *p*-distances data are shown in Suppl. material 1.

## Taxon treatments

### Boiga stoliczkae

Wall, 1909

12D0BFF2-5CDB-5C29-A604-107BF954268C

#### Materials

- **Type status:** Other material. **Occurrence:** sex: female; preparations: whole animal (EtOH); disposition: in collection; associatedSequences: GenBank: PP431561; occurrenceID: 3CD0C420-5333-56B1-887B-97F32F48A29D; **Taxon:** scientificName: *Boigastoliczkae*; nameAccordingTo: Ferdinand Stoliczka; order: Squamata; family: Colubridae; genus: Boiga; taxonRank: species; **Location:** higherGeography: West China; country: China; countryCode: CN; stateProvince: Tibet; county: Gyirong; locality: Rexo Village; verbatimLocality: Gyirong County, Tibet, China; verbatimElevation: 1958 m; **Identification:** identifiedBy: Shiyang Weng; dateIdentified: 31 August 2023; **Event:** eventTime: 2023; **Record Level:** type: PhysicalObject

#### Description

**Figure 1.**
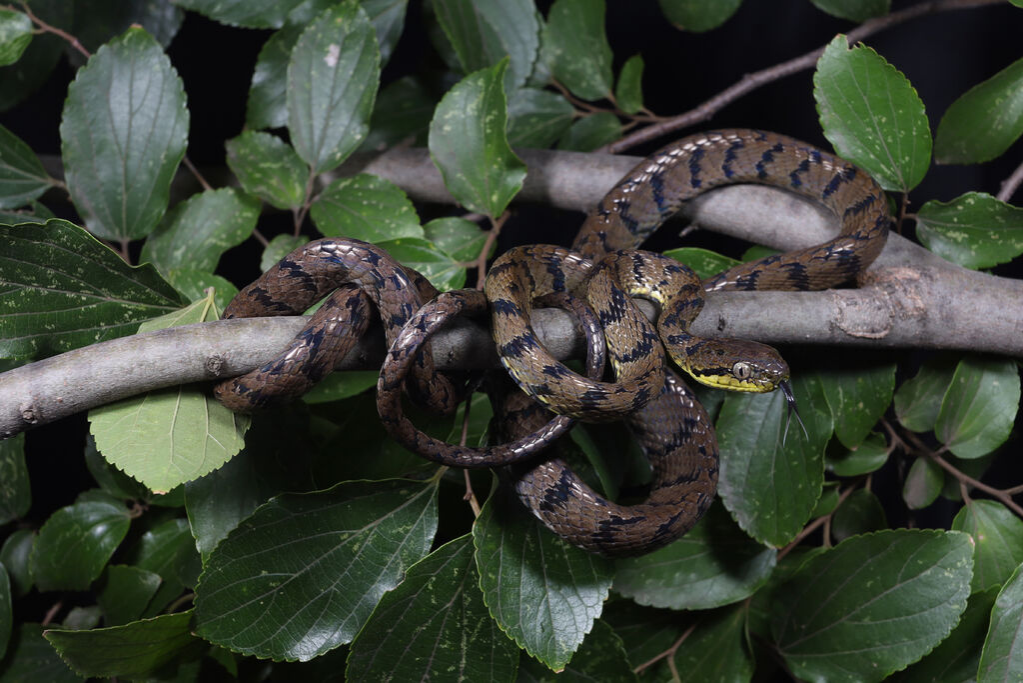
General view of the *Boigastoliczkae* (TBR2023045) in life (photographed by Diancheng Yang).

**Figure 2.**
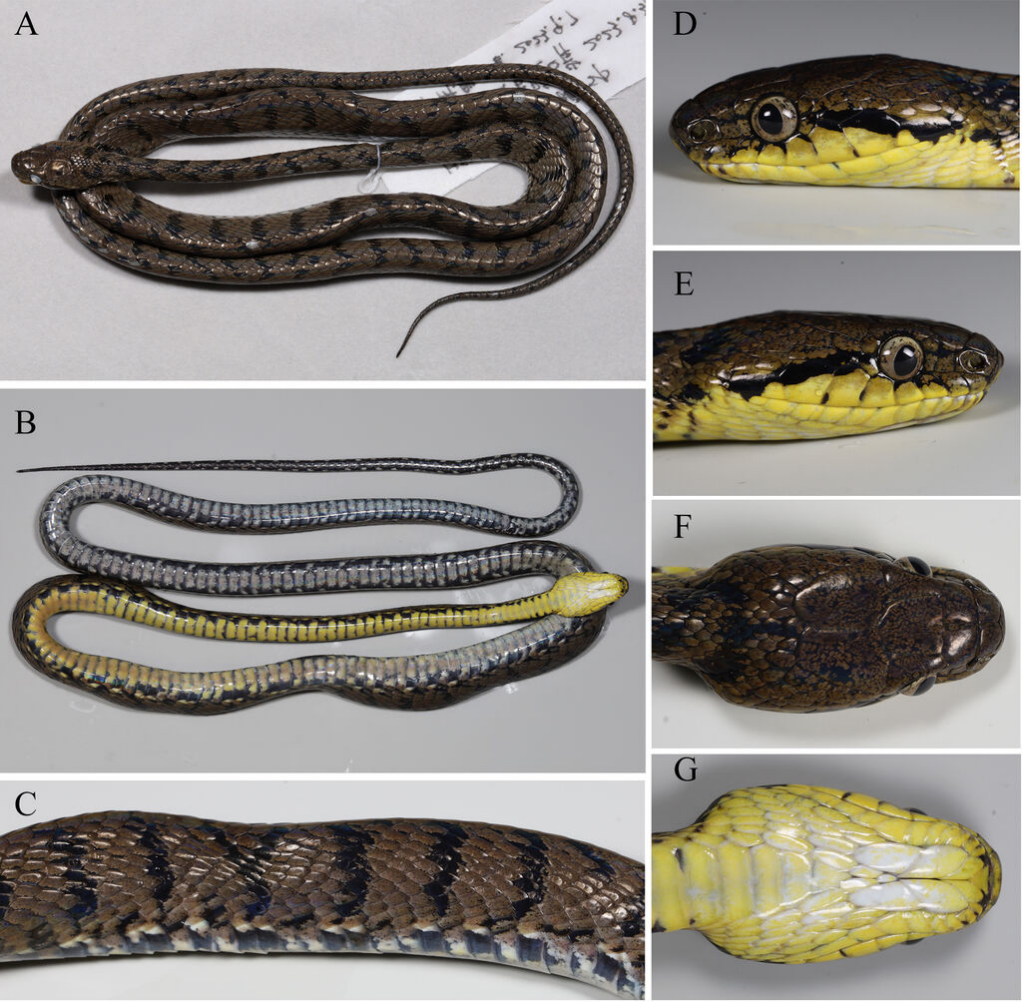
*Boigastoliczkae* (TBR2023045), dorsal (A), ventral (B) and the lateral view of dorsal body (C) views in preservative; right (D), left (E), dorsal (F) and ventral (G) views of the head in life.

An adult female (Figs 1, 2), indicated by the absence of hemipenis. Body slender, total length 1117 mm (SVL 896 mm and TAL 221 mm); tail long, TAL/SVL 24.7%. Head nearly trapezoidal, distinct from neck, dorsally covered with large head scales, head length 24.39 mm; head width 14.71 mm; eye diameter 3.90 mm.

Rostral subtriangular in frontal view, visible from above. Internasals paired, nearly trapezoidal, narrowing anteriorly. Prefrontals paired, more or less quadrangular, wider than long, in contact with loreal. Frontal shield-shaped, nearly straight anteriorly, pointed backwards, slightly longer than wide, supraocular 1/1, much longer than wide; parietals paired. Nasals nearly pentagonal, completely divided by nostril, lower and upper sutures clearly visible, nostril central. Loreal 1/1, nearly square; pre-ocular 1/1, much higher than wide; postocular 2/2, upper one slightly larger than lower one. Temporals 2+2+3, two anterior temporals elongated, the upper one smaller in contact with parietal, the lower one in contact with sixth and seventh supralabial; two middle temporals, smaller than anterior temporal; the lower anterior temporal fused with the middle temporal on the right; three posterior temporals. Supralabials 8/8, 1–2 contacting the nasal, 3^th^ to 5^th^ contacting the orbit, 7^th^ largest. One mental. Infralabials 11/13, the first pair in contact blocking the mental from contacting an anterior pair of chin shields.

Two pairs of chin shields. First five on left and first six on right infralabials touching the first pair of chin shields.

Dorsal scales smooth, rhomboid, imbricate, in 23–21–15 longitudinal rows. Vertebral hexagonal and distinctly enlarged, outermost dorsal scale row on both sides smooth and not enlarged. Ventrals 228 (+ 3 preventrals), subcaudals 98 paired, ventral and subcaudal scales strongly angulated laterally, cloacal plate entire.

**Colouration in life.** The colouration of the specimen's dorsal surface in life is dark brown, with a black stripe distributed almost evenly across the back. The anterior half of the ventral is yellow, while the posterior half gradually becomes white, the edge of the abdomen having small black dots that appear to form a black dotted line when viewed as a whole. The sides of the head have one lateral postorbital stripe, which extends from the last supralabial to the postocular. The pupil is elliptical and black and the sclera is light brown.

**Colouration in alcohol.** The colouration remains similar to the living specimen, but he anterior half of the ventral gradually turning grayish-white, and the black stripe on the dorsal fades to a yellowish-brown hue.

#### Diagnosis

*B.stoliczkae* can be differentiated from its congeners by the following combination of morphological characters: 1) mid-dorsum scale rows 21, all dorsal scales smooth; 2) tail length comparatively long, TaL/SVL 24.7-30.5%; 3) supraocular 1; 4) preocular 1; 5) postocular 2; 6) supralabials 8, 1–2 contacting the nasal, 3^th^ to 5^th^ contacting the orbit, 7^th^ largest; 7) infralabials 10-14; 8) Ventrals 222-247; 9) subcaudal paired, 93-120; 10) cloacal plate entire.

#### Distribution

**Figure 3.**
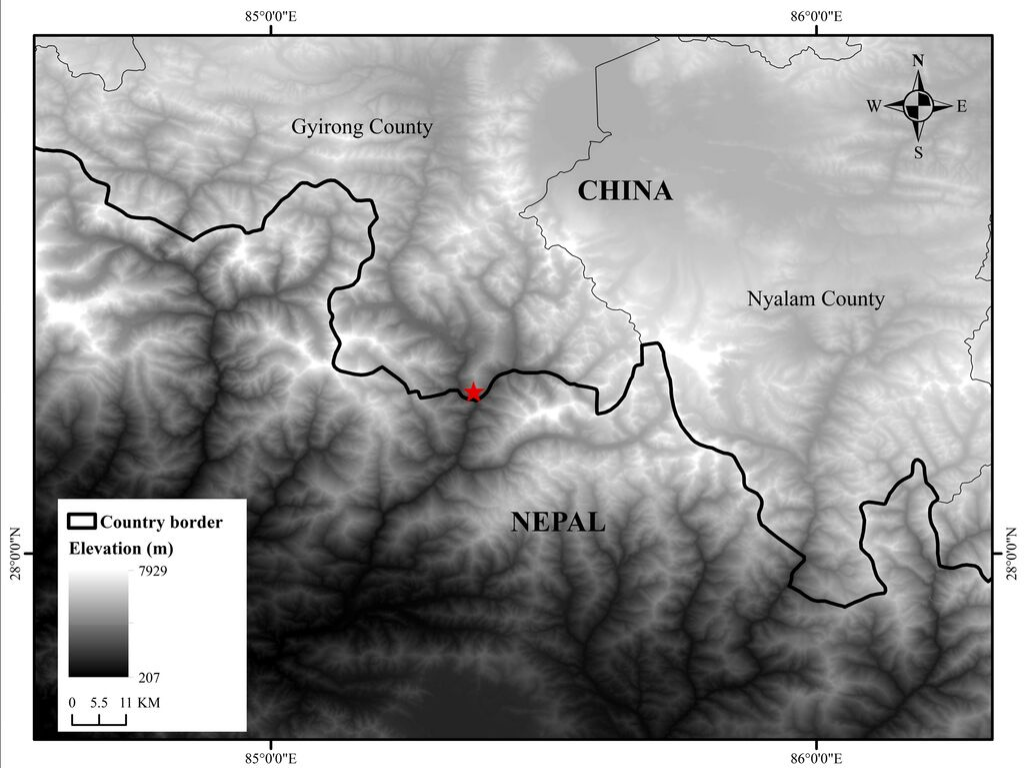
Sample locality (red star) of *Boigastoliczkae* from Gyirong County, Tibet, China.

*B.stoliczkae* is currently known from central Nepal and south of Tibet, China (Fig. 3) through Bhutan to north-eastern India.

#### Ecology

**Figure 4.**
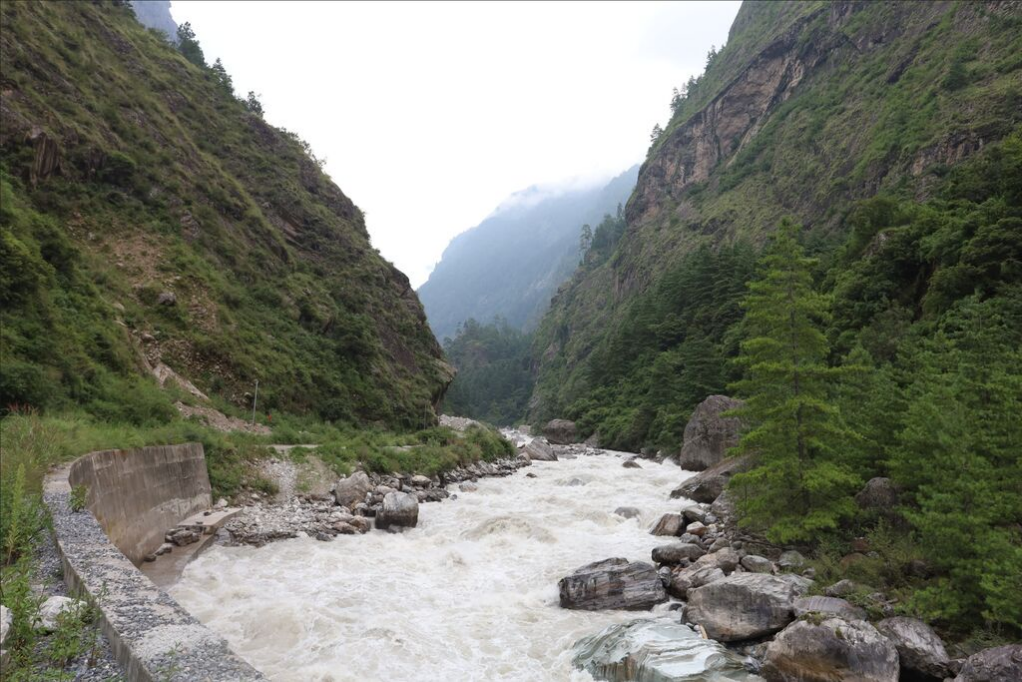
Habitat of *Boigastoliczkae*, Gyirong Town, Gyrong County, Tibet (photographed by Diancheng Yang).

*B.stoliczkae* is mostly semi-arboreal, crepuscular and nocturnal. It is known to inhabit open forest types, agricultural land and human habitations. In our case, the species was found in a road drainage ditch adjacent to the forest edge (Fig. 4).

## Analysis

In order to find the molecular systematic position of the snake in this study, we retrieved cyt b sequences from GenBank for 27 taxa of *Boiga* and used *Telescopusbeetzi* as the outgroup. The alignment for cyt b was 1015 bp in length.

**Figure 5.**
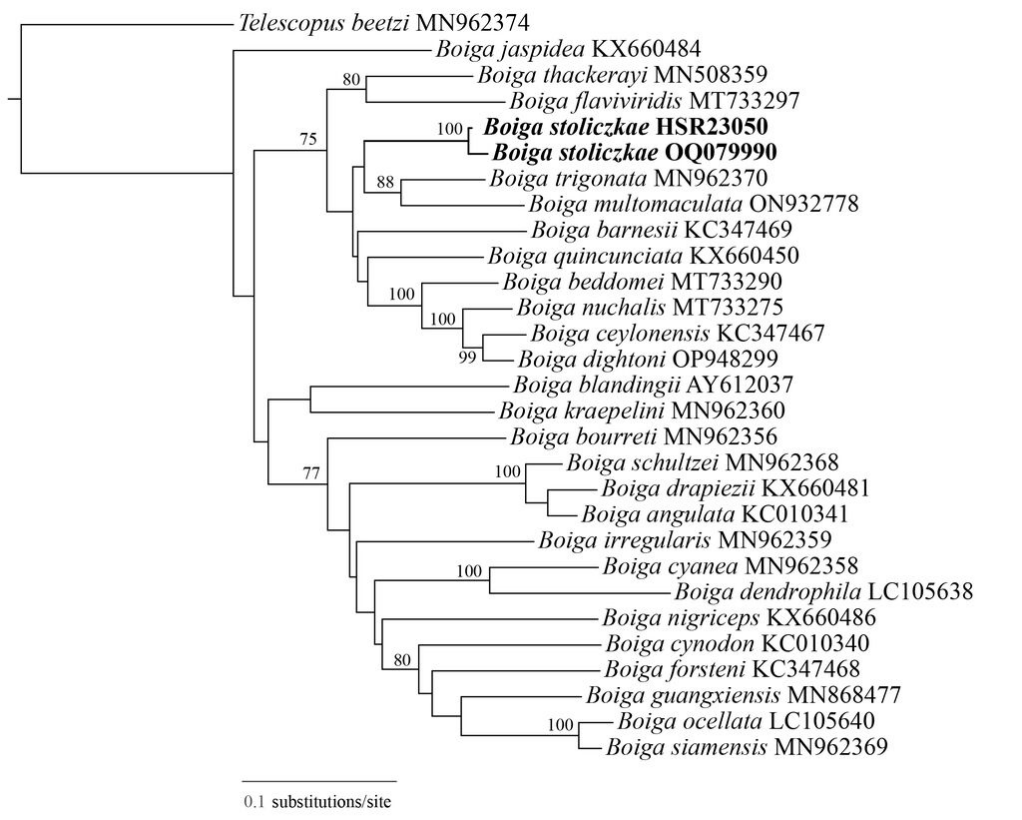
Maximum Likelihood (ML) tree inferred from cyt b. Numbers indicate bootstrap support estimated using Maximum Likelihood (1000 pseudoreplicates) analyses. Maximum Likelihood bootstrap supports (BS) are shown at each internal branch. Posterior probability values < 0.70 are not shown.The new record species in the present study are shown in bold.

Maximum Likelihood (ML) methods were used to reconstruct phylogenetic trees (Fig. 5). The phylogenetic analysis suggested that this specimen was nested in the genus *Boiga* and formed a monophyletic clade with *B.stoliczkae* obtained from Tibet, China.

Genetic distance on cyt b between the specimen of the *B.stoliczkae* collected from Tibet, China and from GenBank sequence was 1.7%, which is substantially lower than interspecific genetic distances of others (11.5-19.5%) (Suppl. material 1).

## Discussion

In the genus *Boiga*, intraspecific colouration and pattern polymorphism are known in *B.forsteni* (Duméril, Bibron & Duméril, 1854), *B.multomaculata*, *B.irregularis* (Bechstein, 1802), *B.drapiezii* (Boie, 1827) and *B.dightoni* (Boulenger, 1894) (Mohapatra et al. 2009, Weinell et al. 2021, Köhler et al. 2023, Narayanan et al. 2023). Previously, only a unicoloured morph was known in *B.stoliczkae*. The dorsal ground colour may vary from cinnamon or cinnamon-rufous to tawny. In this study, we collected the specimen of *B.stoliczkae*, the dorsal ground colour of which is dark brown, with a black stripe distributed almost evenly across the tail. Our results demonstrate that *B.stoliczkae* is polymorphic in colouration and pattern. The research indicates that colour polymorphism, defined as the co-existence of two or more distinct colour variants (morphs) within a population, constitutes a crucial aspect of phenotypic diversity and plays a significant role in enhancing survival and reproductive success (Tang et al. 2023).

In China, five species of the genus *Boiga* have been recorded. Our study reveals that the specimen we collected is the first record of this species in China, thereby broadening its known distribution. Over the years, new reptile species have continually been discovered in the Himalayan Region (Kai et al. 2020, Jiang et al. 2023). The Himalayan Region encompasses multiple countries and poses many access challenges, making comprehensive reptile surveys across the entire region difficult. These challenges have hindered the discovery of new species, further proving that the biodiversity of the Himalayan Region has been underestimated（Xu et al. 2020）.

The Himalaya Region is considered a biodiversity hotspot due to its unique geographic and climatic conditions, which have led to the evolution of a diverse range of plant and animal species (Xu et al. 2020). As fieldwork continues, more and more new species and records of species are being reported, which indirectly proves that there are still species awaiting discovery in the Himalayan Region. Continued biodiversity monitoring and increased attention are still needed.

## Supplementary Material

XML Treatment for Boiga stoliczkae

1AC1A5B0-774B-5EE5-AB34-87D8E69D0354 10.3897/BDJ.12.e123669.suppl1 Supplementary material 1 Uncorrected *p*-distances (%) amongst the *Boiga* species, based on partial mitochondria cytb gene. Data type genomic File: oo_1006130.docx https://binary.pensoft.net/file/1006130 Shiyang Weng, Dian-Cheng Yang, Cong Wei, Peng Li, Zhangbo Cui, ZhiHao Jiang, Song Huang

## References

[B11236379] Burbrink Frank T, Lawson Robin, Slowinski Joseph B (2000). Mitochondrial DNA phylogeography of the polytypic North American rat snake (*Elapheobsoleta*): a critique of the subspecies concept. Evolution.

[B11236820] Burland T. G. (2000). DNASTAR's Lasergene sequence analysis software. Methods in Molecular biology.

[B11236503] Darko Yaa, Voss Olaf, Uetz Peter (2022). A dictionary of abbreviations used in reptile descriptions. Zootaxa.

[B11236477] Dowling Herndon Glenn (1951). A proposed standard system of counting ventrals in snakes. British Journal of Herpetology.

[B11236494] Dowling H. G. (1951). A proposed method of expressing scale reductions in snakes. Copeia.

[B11744774] Ganesh S. R., Achyuthan N. S., Chandramouli S. R., Vogel Gernot (2020). Taxonomic revision of the Boiga ceylonensis group (Serpentes: Colubridae): re-examination of type specimens, redefinition of nominate taxa and an updated key. Zootaxa.

[B11744741] Giri Varad, Deepak V., Tillack Frank, Pawar Swapnil (2019). A New Species of Boiga Fitzinger, 1826 (Serpentes: Colubridae) from The Northern Western Ghats of India. Journal of the Bombay Natural History Society.

[B11236355] Huang Junkai, Yang Yan, Wu Yunke, Li Ke, Hang Guohui, Yuan Zhiyong (2021). Discovery of *Boigasiamensis* Nutaphand, 1971 (Squamata, Colubridae) from Yunnan Province, China. Russian Journal of Herpetology.

[B11236486] Huang Song (2021). Sinoophis.

[B11740423] Jiang Jianping, Cai Bo, Wang Bin, Chen Weitao, Wen Zhixin, Zhang Dezhi, Sui Lulu, Ma Shun (2023). New vertebrate species discovered in China in 2022. Biodiversity Science.

[B11740407] Kai Wang, Jinlong Ren, Hongman Chen, Zhitong Lyu, Xianguang Guo, Ke Jiang, Jinmin Chen, Jiatang Li, Peng Guo, Yingyong Wang, Jing Che (2020). The updated checklists of amphibians and reptiles of China. Biodiversity Science.

[B11772779] Kalyaanamoorthy Subha, Minh Bui Quang, Wong Thomas K F, von Haeseler Arndt, Jermiin Lars S (2017). ModelFinder: fast model selection for accurate phylogenetic estimates. Nature Methods.

[B11236463] Köhler Gunther, Charunrochana Panupong Thammachoti, Mogk Linda, Than Ni Lar, Kurniawan Nia, Kadafi Ahmad Muammar, Das Abhijit, Tillack Frank, O’Shea Mark (2023). A taxonomic revision of *Boigamultomaculata* (Boie, 1827) and *B.ochracea* (Theobald, 1868), with the description of a new subspecies (Squamata, Serpentes, Colubridae). Zootaxa.

[B11236454] Kramer E (1977). Zur Schlangenfauna Nepals. Revue Suisse de Zoologie.

[B11236525] Mohapatra Pratyush, Das Abhijit, Tillack Frank, Dutta Sushil (2009). Taxonomy, Natural History and distribution of *Boigaforsteni* (Dumeril, Bibron et Dumeril, 1854) (Serpentes: Colubridae) from Orissa, India. Russian Journal of Herpetology.

[B11236512] Narayanan Surya, Das Sandeep, Anvar Y., Tillack Frank, Mohapatra Pratyush, Gower David, Rajkumar K. P., Deepak V. (2023). On the taxonomic validity of *Boigawhitakeri* Ganesh et al., 2021 with new insights on *Boigadightoni* (Boulenger, 1894) (Reptilia: Squamata: Colubridae). Vertebrate Zoology.

[B11236446] Smith M A (1944). The fauna of British India, Ceylon and Burma, including the whole of the Indo-Chinese sub-region. Reptilia and Amphibia. 3 (Serpentes).

[B11236401] Tamura Koichiro, Stecher Glen, Kumar Sudhir (2021). MEGA11: Molecular Evolutionary Genetics Analysis Version 11. Molecular Biology and Evolution.

[B11739412] Tang Chen-Yang, Zhang Xiaohu, Xu Xiao, Sun Shijie, Peng Changjun, Song Meng-Huan, Yan Chaochao, Sun Huaqin, Liu Mingfeng, Xie Liang, Luo Shu-Jin, Li Jia-Tang (2023). Genetic mapping and molecular mechanism behind color variation in the Asian vine snake.. Genome biology.

[B11236419] Uetz Peter, Freed P, Aguilar R, Kudera J, Reyes F, Hošek J (2024). The Reptile Database. http://www.reptile-database.org/.

[B11236437] Wall F. (1909). Remarks on some forms of *Dipsadomorphus*. Records of the Zoological Survey of India.

[B11236366] Weinell Jeffrey L, Barley Anthony J, Siler Cameron D, Orlov Nikolai L, Ananjeva Natalia B, Oaks Jamie R, Burbrink Frank T, Brown Rafe M (2021). Phylogenetic relationships and biogeographic range evolution in cat-eyed snakes, *Boiga* (Serpentes: Colubridae). Zoological Journal of the Linnean Society.

[B11739335] Xu Wei, Dong Wen-Jie, Fu Ting-Ting, Gao Wei, Lu Chen-Qi, Yan Fang, Wu Yun-He, Jiang Ke, Jin Jie-Qiong, Chen Hong-Man, Zhang Ya-Ping, Hillis David M, Che Jing (2020). Herpetological phylogeographic analyses support a Miocene focal point of Himalayan uplift and biological diversification. National Science Review.

[B11236429] Zhao Er Mi (2006). Snakes of China.

