# Supplementary material for: A new record and a novel morph description of Boigastoliczkae (Squamata, Colubridae) from China
Source: Biodivers Data J. 2024 Jul 4;12:e123669. doi: 10.3897/BDJ.12.e123669 (PMC11250174; doi:10.3897/BDJ.12.e123669)
Supplement: Supplementary material 1 — Uncorrected p-distances (%) amongst the Boiga species, based on partial mitochondria cytb gene. [file bdj-12-e123669-s001.docx]

**Table 1.** Uncorrected p-distances (%) among the *Boiga* species based on partial mitochondria cytb gene.

|  |  | 1 | 2 | 3 | 4 | 5 | 6 | 7 | 8 | 9 | 10 | 11 | 12 | 13 | 14 | 15 | 16 | 17 | 18 | 19 | 20 | 21 | 22 | 23 | 24 | 25 | 26 | 27 | 28 |
| --- | --- | --- | --- | --- | --- | --- | --- | --- | --- | --- | --- | --- | --- | --- | --- | --- | --- | --- | --- | --- | --- | --- | --- | --- | --- | --- | --- | --- | --- |
| 1 | HSR23050 | – |  |  |  |  |  |  |  |  |  |  |  |  |  |  |  |  |  |  |  |  |  |  |  |  |  |  |  |
| 2 | ***B. stoliczkae*** | 1.7 | – |  |  |  |  |  |  |  |  |  |  |  |  |  |  |  |  |  |  |  |  |  |  |  |  |  |  |
| 3 | *B. angulata* | 17.7 | 17.9 | – |  |  |  |  |  |  |  |  |  |  |  |  |  |  |  |  |  |  |  |  |  |  |  |  |  |
| 4 | *B. barnesii* | 13.4 | 13.7 | 16.4 | – |  |  |  |  |  |  |  |  |  |  |  |  |  |  |  |  |  |  |  |  |  |  |  |  |
| 5 | *B. beddomei* | 12.4 | 12.1 | 16.3 | 13.6 | – |  |  |  |  |  |  |  |  |  |  |  |  |  |  |  |  |  |  |  |  |  |  |  |
| 6 | *B. blandingii* | 17.3 | 17.4 | 17.7 | 16.6 | 16.3 | – |  |  |  |  |  |  |  |  |  |  |  |  |  |  |  |  |  |  |  |  |  |  |
| 7 | *B. bourreti* | 18.1 | 17.7 | 16.2 | 16.6 | 16.2 | 17.1 | – |  |  |  |  |  |  |  |  |  |  |  |  |  |  |  |  |  |  |  |  |  |
| 8 | *B. ceylonensis* | 13.4 | 13.7 | 17.0 | 13.9 | 9.4 | 17.7 | 16.3 | – |  |  |  |  |  |  |  |  |  |  |  |  |  |  |  |  |  |  |  |  |
| 9 | *B. cyanea* | 17.2 | 17.5 | 15.1 | 17.0 | 17.7 | 17.5 | 15.6 | 18.3 | – |  |  |  |  |  |  |  |  |  |  |  |  |  |  |  |  |  |  |  |
| 10 | *B. cynodon* | 17.7 | 17.5 | 16.1 | 18.5 | 17.6 | 17.2 | 15.6 | 18.8 | 15.5 | – |  |  |  |  |  |  |  |  |  |  |  |  |  |  |  |  |  |  |
| 11 | *B. dendrophila* | 19.0 | 19.0 | 16.2 | 17.7 | 17.5 | 18.6 | 15.8 | 18.4 | 13.0 | 17.0 | – |  |  |  |  |  |  |  |  |  |  |  |  |  |  |  |  |  |
| 12 | *B. dightoni* | 12.5 | 12.7 | 17.0 | 13.0 | 9.3 | 15.9 | 15.2 | 4.8 | 16.8 | 18.8 | 17.0 | – |  |  |  |  |  |  |  |  |  |  |  |  |  |  |  |  |
| 13 | *B. drapiezii* | 18.2 | 18.1 | 4.9 | 16.7 | 16.0 | 18.0 | 15.7 | 17.3 | 15.7 | 16.9 | 16.7 | 17.1 | – |  |  |  |  |  |  |  |  |  |  |  |  |  |  |  |
| 14 | *B. flaviviridis* | 14.1 | 14.2 | 17.2 | 15.7 | 13.4 | 17.1 | 17.5 | 13.1 | 17.6 | 19.2 | 19.6 | 13.1 | 17.1 | – |  |  |  |  |  |  |  |  |  |  |  |  |  |  |
| 15 | *B. forsteni* | 18.3 | 18.8 | 15.3 | 18.5 | 17.1 | 15.8 | 14.3 | 17.7 | 14.7 | 15.0 | 16.8 | 16.2 | 16.9 | 17.8 | – |  |  |  |  |  |  |  |  |  |  |  |  |  |
| 16 | *B. guangxiensis* | 17.8 | 18.1 | 16.2 | 16.4 | 16.2 | 16.4 | 15.7 | 17.0 | 16.8 | 14.8 | 17.0 | 16.1 | 16.1 | 16.8 | 13.5 | – |  |  |  |  |  |  |  |  |  |  |  |  |
| 17 | *B. irregularis* | 16.8 | 16.7 | 14.5 | 17.5 | 17.1 | 17.2 | 15.2 | 17.3 | 15.7 | 15.1 | 15.7 | 17.5 | 15.5 | 17.2 | 14.5 | 15.1 | – |  |  |  |  |  |  |  |  |  |  |  |
| 18 | *B. jaspidea* | 17.1 | 17.2 | 17.3 | 16.9 | 17.2 | 17.0 | 16.6 | 18.5 | 18.2 | 17.5 | 20.3 | 18.3 | 18.6 | 16.3 | 16.7 | 16.9 | 17.5 | – |  |  |  |  |  |  |  |  |  |  |
| 19 | *B. kraepelini* | 17.1 | 16.8 | 15.7 | 16.2 | 14.5 | 14.6 | 15.7 | 15.3 | 16.6 | 17.6 | 17.5 | 14.9 | 16.5 | 16.5 | 15.8 | 17.0 | 16.0 | 16.3 | – |  |  |  |  |  |  |  |  |  |
| 20 | *B. multomaculata* | 12.9 | 13.4 | 16.9 | 14.7 | 11.7 | 16.3 | 16.3 | 13.2 | 17.0 | 18.9 | 19.3 | 13.5 | 17.9 | 13.7 | 16.8 | 17.5 | 16.0 | 17.1 | 15.4 | – |  |  |  |  |  |  |  |  |
| 21 | *B. nigriceps* | 18.0 | 17.5 | 16.9 | 18.2 | 17.6 | 17.6 | 14.9 | 17.5 | 16.1 | 15.6 | 16.6 | 14.0 | 17.3 | 17.1 | 16.0 | 14.9 | 15.9 | 17.1 | 17.9 | 16.8 | – |  |  |  |  |  |  |  |
| 22 | *B. nuchalis* | 13.5 | 13.4 | 16.6 | 13.6 | 8.5 | 16.5 | 15.8 | 6.7 | 18.2 | 19.0 | 19.2 | 5.8 | 17.3 | 13.1 | 16.7 | 17.1 | 18.1 | 17.7 | 14.6 | 13.6 | 16.9 | – |  |  |  |  |  |  |
| 23 | *B. ocellata* | 18.6 | 18.5 | 16.1 | 19.0 | 16.0 | 16.4 | 17.1 | 17.5 | 16.1 | 15.6 | 15.3 | 17.0 | 16.8 | 18.0 | 14.6 | 12.7 | 16.2 | 18.3 | 17.0 | 17.0 | 15.9 | 17.6 | – |  |  |  |  |  |
| 24 | *B. quincunciata* | 11.8 | 12.2 | 15.8 | 14.1 | 11.9 | 15.7 | 16.0 | 12.2 | 18.0 | 18.0 | 19.2 | 12.1 | 16.6 | 13.0 | 17.2 | 17.0 | 17.0 | 17.1 | 15.3 | 11.6 | 16.9 | 11.6 | 17.5 | – |  |  |  |  |
| 25 | *B. schultzei* | 18.1 | 18.4 | 5.5 | 17.9 | 17.4 | 17.5 | 16.5 | 17.4 | 15.8 | 17.0 | 16.7 | 17.9 | 6.0 | 17.8 | 17.0 | 16.1 | 15.3 | 18.2 | 16.5 | 17.3 | 16.7 | 18.1 | 16.8 | 16.2 | – |  |  |  |
| 26 | *B. siamensis* | 19.5 | 19.7 | 16.2 | 19.0 | 16.6 | 15.8 | 16.5 | 16.7 | 15.9 | 15.2 | 15.3 | 17.1 | 16.6 | 18.2 | 13.7 | 12.5 | 15.8 | 17.9 | 17.2 | 16.2 | 16.1 | 17.7 | 3.7 | 17.9 | 16.3 | – |  |  |
| 27 | *B. thackerayi* | 12.5 | 13.0 | 16.5 | 14.6 | 13.4 | 16.4 | 18.0 | 14.6 | 16.6 | 19.0 | 18.8 | 13.9 | 17.1 | 12.2 | 17.6 | 17.5 | 18.7 | 15.7 | 16.1 | 14.4 | 17.3 | 13.4 | 17.8 | 10.9 | 17.2 | 17.9 | – |  |
| 28 | *B. trigonata* | 11.5 | 11.8 | 16.6 | 13.6 | 11.6 | 16.5 | 16.1 | 13.1 | 16.5 | 18.4 | 17.6 | 12.1 | 16.9 | 14.2 | 18.1 | 16.8 | 17.6 | 17.4 | 15.3 | 10.9 | 17.3 | 11.7 | 17.2 | 11.9 | 17.6 | 17.2 | 13.1 | – |
